# Extrapolating Antibiotic Sales to Number of Treated Animals: Treatments in Pigs and Calves in Switzerland, 2011–2015

**DOI:** 10.3389/fvets.2019.00318

**Published:** 2019-09-20

**Authors:** Rosa Stebler, Luís P. Carmo, Dagmar Heim, Hanspeter Naegeli, Klaus Eichler, Cedric R. Muentener

**Affiliations:** ^1^Winterthur Institute of Health Economics, Zurich University of Applied Sciences, Zurich, Switzerland; ^2^Swissmedic, Swiss Agency for Therapeutic Products, Berne, Switzerland; ^3^Vetsuisse, Veterinary Public Health Institute, University of Bern, Bern, Switzerland; ^4^Federal Food Safety and Veterinary Office, Bern, Switzerland; ^5^Institute of Veterinary Pharmacology and Toxicology, Vetsuisse, University of Zurich, Zurich, Switzerland

**Keywords:** antibiotics, antimicrobial consumption, course dose, pigs, calves

## Abstract

To evaluate the contribution of antimicrobial use in human and veterinary medicine to the emergence and spread of resistant bacteria, the use of these substances has to be accurately monitored in each setting. Currently, various initiatives collect sales data of veterinary antimicrobials, thereby providing an overview of quantities on the market. However, sales data collected at the level of wholesalers or marketing authorization holders are of limited use to associate with the prevalence of bacterial resistances at species level. We converted sales data to the number of potential treatments of calves and pigs in Switzerland for the years 2011 to 2015 using animal course doses (ACD). For each authorized product, the number of potential therapies was derived from the sales at wholesaler's level and the ACD in mg per kg. For products registered for use in multiple species, a percentage of the sales was attributed to each authorized species according to their biomass distribution. We estimated a total of 5,914,349 therapies for pigs and 1,407,450 for calves in 2015. Using the number of slaughtered animals for that year as denominator, we calculated a treatment intensity of 2.15 therapies per pig and 5.96 per calf. Between 2011 and 2015, sales of veterinary antimicrobials decreased by 30%. The calculated number of potential therapies decreased by 30% for pigs and 15% for calves. An analysis of treatment intensity at antimicrobial class level showed a decrease of 64% for colistin used in pigs, and of 7% for macrolides used in both pigs and calves. Whereas the use of 3rd and 4th generation cephalosporins in calves decreased by 15.8%, usage of fluoroquinolones increased by 10.8% in the same period. Corresponding values for pigs were −16.4 and +0.7%. This is the first extrapolation of antimicrobial usage at product level for pigs and calves in Switzerland. It shows that calves were more frequently treated than pigs with a decreasing trend for both number of therapies and use of colistin, macrolides and cephalosporins 3rd and 4th generations. Nonetheless, we calculated an increase in the usage of fluoroquinolones. Altogether, this study's outcomes allow for trend analysis and can be used to assess the relationship between antimicrobial use and resistance at the national level.

## Introduction

Use of antimicrobials contributes to the emergence and spread of resistant bacteria in both humans and animals. As early as in the 1960's concerns arose in relation to therapeutic, preventive and growth-promoting treatments in food-producing animals. The fact that most antibiotic classes are administered to treat infections in both humans and animals was one of the major concerns (1, 2). Monitoring antimicrobial usage is therefore a prerequisite to assess the impact of antibiotic treatments on the selection and spread of bacterial resistances. In order to achieve that goal, a number of programs monitoring sales and/or usage of antimicrobials have been established both at national level, for example in Switzerland [ARCH-Vet; (3)], Denmark (4), and international level [ESVAC project of the European Medicines Agency; (5)]. These programs do not only aim at the identification of trends in sales and usage of antimicrobial classes but should also allow establishing a link with changes observed in resistance monitoring programs, thereby providing a basis for risk assessment and evaluation of regulatory interventions (6). In order to assess the association between antimicrobial use and resistance, it is of crucial relevance to obtain consumption data at species or, when possible, production type level; there are several species and production type-specific factors that can impact on the relationship between use and resistance. Those factors include age at treatment, age and weight at slaughter, products available per species or production type, and especially production structures (7–9).

Antimicrobial sales data are defined as the minimal standard for monitoring programs by the World Organization for Animal Health [Office International des Epizooties, OIE; (10)]. They can be collected at either the manufacturer, wholesaler or pharmacy level depending on the national distribution routes of the products. Sales data are useful to evaluate long-term trends but do not include information about dose, route of administration, indication or duration of therapy. However, in the context of resistance epidemiology, only data about actual use of antimicrobials collected either at prescription or patient level might deliver the information necessary to establish and evaluate implemented measures. Such data can only be currently collected in few countries with advanced collection systems, such as Denmark (4) and The Netherlands (11) among other European countries. The AACTING network is maintaining a list of the various collection systems already in place (www.aacting.org). The collection of data at animal level is the ultimate goal of antimicrobial monitoring systems and, until this is available in all participating countries, alternatives using normalization of sales data by the total weight of the food producing animal population as a denominator have been developed. One such denominator is the population correction unit of the ESVAC project (12). Other institutions (13) and countries, including Canada (14) and Switzerland [ARCH-Vet; (3)], have implemented similar methods in their surveillance systems. As usage of antimicrobials is strongly dependent on population structure and repartition between high and low-using species, the normalization by weight may provide information on long-term trends but at the same time, a higher usage in one species will be “diluted” by lower usage species/production types (like dairy cows) with a large contribution to the overall livestock biomass (15). It is therefore important to measure antibiotic consumption as near as possible to the end users, i.e., to obtain information on species, dosage, duration and whenever possible, indication. The extrapolation of sales data using course doses is an interim measure to data collection at animal level. Course dose indicators have been proposed, such as the Animal Course Dose (ACD) by the French Agency for Food, Environmental, and Occupational Health & Safety (16) or Defined Course Dose (DCDvet) from the EMA (17). An advantage of ACD is its product-specific calculation, therefore better representing national specificities than DCDvet units. ACDs are established for each product using data from the summary of product characteristics (SPC) and contain the necessary detail on both dose and therefore potency, and duration of use.

The main aim of this study was to provide for the first time an extrapolation of the available national sales data to the number of treated animals in Switzerland. We chose to specifically investigate the treatment of pigs and calves because these are mainly reared and treated in groups via oral application. Due to the lack of detailed data about repartition of sales, we made assumptions regarding weight at treatment and repartition of sales data between species using a previously published repartition method. We then defined ACD for each product containing antimicrobials authorized in Switzerland for use in either pigs or calves and combined this information with national antibiotic sales data to extrapolate the number of potentially treated animals during the years 2011 to 2015.

## Materials and Methods

### Data Collection

Veterinary antibiotic sales data for the years 2011 to 2015 were obtained from the Federal Office of Food Safety under a confidentiality agreement. Since 2004, sales data are collected in Switzerland from marketing authorization holders based on Article 35 of the Ordinance of Veterinary Medicines (18). Marketing authorization holders are required to deliver data on every product containing antimicrobials that was sold during a calendar year. Products subject to data collection are defined by their ATCvet codes (19) as listed in the ESVAC project (12). Additionally, data on antibiotic products not considered by the ESVAC project, like sprays or products to treat sensory organs, are also collected. Data obtained from the Federal Office of Food Safety for this study contained the quantity of active antimicrobial ingredient sold in kilogram for each product and year under investigation.

### Animal Populations, Animal Weights, and Species Repartition

The amount of antimicrobials sold of products authorized for a single species was directly assigned to their target species. For each product authorized for more than one species, a repartition had to be determined. We used two distinct methods: the first one was used for premixes, the latter being legally defined in Switzerland as being “veterinary medicinal products used to treat groups of animals and incorporated into either water or feed” [Ordinance on authorizations for medicinal products, Art. 2; (20)]. For all of these products, periodic safety update reports (PSURs) containing data on species repartition submitted to Swissmedic, the Swiss Agency for Therapeutic Products, during the years 2007 to 2012, were used. As premixes represented only 28 products from a total of 112 under investigation but between 57.6% (2015) and 67.8% (2011) of the total sales, another repartition method had to be used for oral solutions, oral powders and injectables. This repartition was done according to biomass repartition as described by Carmo et al. (21). Briefly, for each product authorized for one or more target species, each target species was assigned a percentage of kg of the total sales representing the proportion of its biomass in the total biomass of the list of authorized species for the product. For the present study, food producing animal population numbers were obtained from the Federal Office of Statistics (www.bfs.admin.ch), number of dogs from the ANIS database (Identitas AG, Bern, www.anis.ch) and the number of cats from the Swiss Association of pet food producers (Verband für Heimtiernahrung, Bern, www.vhn.ch). In analogy with calculations of the population correction unit (PCU) of ESVAC (12), the number of slaughtered animals were used for fattening pigs and calves, whereas data for dairy cows, sows, sheep, goats, horses, dogs, and cats represent live animals. Throughout the text and in the tables, “pigs” refer to fattening pigs.

Supplementary Table 1 lists the number of animals and the weights used for the biomass repartition. The most likely weight at treatment was sourced from the ESVAC report (12, 22). As heavy animals with a rather low treatment intensity, like dairy cows, skew the biomass repartition, we chose to only include them in the calculation when they were either explicitly listed as authorized species (“dairy cows”) or, when a withdrawal time for milk was given in the SPC of the product. For pigs, we did not include the production stage of piglets or weaners. Using the number of animals in different production stages presents some challenges, the most prominent one for pigs being the lack of available data for the repartition of use between piglets and e.g., fattening pigs. Only few antimicrobials are primarily used in piglets or weaners, colistin being such an example. For almost all other products authorized for pigs, no data are available to stratify antimicrobial consumption per different age classes using sales data. Repartition data will only be available once reporting of all treatments with antimicrobials in Switzerland is made mandatory at the end of the year 2019. For this reason and because sales data include the use of antimicrobials by all the age categories of the species for the years under investigation, we used slaughtered numbers of pigs as denominator for the therapeutic incidence in this species. Finally, for injectable products authorized without indication of the production stage (“bovines” containing dairy cows and “pigs” representing slaughtered pigs and sows) we used raw data provided by experts for the study by Carmo et al. (23) to determine if a use would take place in the particular production stage under consideration.

### Calculation of Animal Course Doses and Therapeutic Intensity

The Animal Course Dose (ACD) was calculated for each active pharmaceutical ingredient contained in each product authorized during the years under investigation. Data were collected from the authorized summary of product characteristics (24) and entered into an MS Excel sheet containing: name of the product, authorization number, list of authorized species, active ingredient(s), dose and duration. Doses given in international units were converted to mg using conversion factors listed in the ESVAC report (12). Whenever the recommended dose was a range, the highest recommended dose and longest duration were chosen to reflect the minimal number of animals potentially treated. Moreover, when different doses were authorized for different indications, the most likely indication was chosen. This was the case for products presenting both a prophylactic or metaphylactic indication with different doses and duration. ACDs were defined per kg and the ACD per animal obtained by multiplication with the likely weight at treatment. To take Swiss specificities into account, the weight at treatment for pigs was taken from a previous study by Schnetzer et al. (25) and the weight for calves based on expert opinion (Prof. M. Kaske, Zurich, personal communication).

Therapeutic intensity reflects the number of ACDs per slaughtered animal (pig or calf) in 1 year. For combination products, the number of ACDs was calculated separately for each active pharmaceutical ingredient. Therefore, a single treatment with a combination containing 3 antimicrobials results in 3 ACDs. ACD and intensity were calculated using the following equations:

$$\begin{array}{l} \;Number\;of\;ACDs=\frac{total\;quantity\;of\;active\;ingredient\;sold\;in\;one\;year\;\left(mg\right)}{daily\;dose\;\left(\frac{mg}{kg}\right)\;\times \;duration\;of\;tratment\;\left(days\right)\;\times \;weight\;at\;treatment\;\left(kg\right)}\\ Therapeutic\;intensity\;in\;species\;X=\;\frac{Number\;of\;ACDs\;in\;species\;X}{Total\;number\;of\;animals\;for\;species\;X} \end{array}$$

## Results

From the year 2011 to 2015, sales of antibiotics for use in food producing animals decreased by 29.8% (Table 1). In the same time, the percentage represented by premixes decreased from 67.8 to 57.7%. Therefore, measured in kg, antimicrobials sold in premixes made the largest part of yearly sales of antimicrobials for the veterinary medicine. As a consequence, pigs and calves are the most pertinent species among food producing animals to be investigated for use and trend detection. In tonnage sold for use in these species, the decrease in the 5 years under investigation is comparable: 38.4% in pigs and 30.1% in calves. However, normalizing these numbers to the respective biomass of the produced (slaughtered) population reveals a much higher use per kg of biomass for calves (449.9 mg/kg biomass in 2015) than for pigs (77.4 mg/kg biomass). The difference between both species even increased from 4.8-fold higher for calves in 2011 to 5.8 in 2015.

**Table 1:** Sales, biomass and mg per kg biomass for food producing animals as well as pigs and calves for the years 2011 to 2015.

	**2011**	**2012**	**2013**	**2014**	**2015**
**ALL FOOD PRODUCING ANIMALS**					
Sales (kg)^a^	58,942	54,169	50,370	46,147	41,378
% Premixes	67.8	65.7	64.5	61.9	57.7
mg/kg	72	66	62	56	51
**PIGS**					
Sales (kg)	22,475	20,276	18,890	16,458	13,845
mg/kg	121.8	112.5	108.1	92.0	77.4
**CALVES**					
Sales (kg)	21,293	19,299	17,941	16,385	14,886
mg/kg	582.1	537.5	508.3	465.3	449.9

a *Sales data detailed by antimicrobial classes for the years under investigation are available under https://www.blv.admin.ch/blv/de/home/tiere/publikationen-und-forschung/statistiken-berichte-tiere.html*.

Normalizing sales data to either the overall biomass of food producing animals or to the biomass of a particular species is a crude estimate of antimicrobials use, not taking dose or duration into account. We therefore calculated the number of course doses (ACDs) per product and species. A summary of the results is presented in Table 2. The total number of ACDs was approximately 4.5 times higher in pigs and decreased by 31.7% over the years under investigation, whereas the decrease for calves was 23.0%. Normalization to the number of slaughtered animals showed a much slower decrease of 14.9% for calves between 2011 and 2015 compared to 29.6% in pigs. As a result, the difference between both species grew from 2.3-fold in the year 2011 to 2.8-fold in the final year under investigation.

**Table 2:** Number of estimated ACDs per pigs or calves, oral and parenteral application, from 2011 to 2015.

	**2011**	**2012**	**2013**	**2014**	**2015**
**PIGS**					
Number of ACDs	8,663,191	7,686,268	7,184,114	6,674,046	5,914,349
Number slaughtered	2,839,106	2,773,726	2,689,576	2,751,721	2,753,256
Intensity^a^	3.051	2.771	2.671	2.425	2.148
**CALVES**					
Number of ACDs	1,828,599	1,687,942	1,636,930	1,521,050	1,407,450
Number slaughtered	261,308	256,471	252,118	251,509	236,343
Intensity^a^	6.998	6.581	6.493	6.048	5.955

a *Number of ACDs per slaughtered animal*.

Not all antibiotics have the same potential impact on resistance selection and consequences for the treatment of both humans and animals. Moreover, different products are authorized for distinct conditions in pigs or calves. The repartition of the number of ACDs per class of antimicrobials was therefore calculated separately for each species for the year 2015. Table 3 presents the repartition by antimicrobial class for ingredients sold in premixes and as parenteral injections. In this year, polymyxins (in form of colistin) were the class with the highest potential numbers of ACDs per pigs, followed by sulfonamides. In calves, the highest number was represented by penicillins (mainly sold as aminopenicillins) followed by tetracyclines. The total number of ACDs per animal was 4.43 times higher in calves than in pigs. The same calculation was done for injectable products as these may contain antimicrobials of the highest priority [HPCIA; (26)] not available for oral application. For pigs, the highest number of ACDs per animal in the year 2015 was represented by macrolides, followed by aminoglycosides and fluoroquinolones. For calves (amino)penicillins were the class with the highest number of course doses per animal, followed by macrolides and aminoglycosides. The total number of potential ACDs per animal for injectable products in the year 2015 was 1.485 for calves and 1.088 for pigs.

**Table 3:** Number of estimated ACDs per animal, antimicrobials administered as premix or parenterally in pigs and calves for the year 2015, presented by classes of antimicrobials.

**Classes of antimicrobials**	**Pigs**		**Calves**	
	**Parenteral**	**Premixes**	**Parenteral**	**Premixes**
Sulfonamides	0.050	0.177	0.042	0.632
Penicillins	0.389	0.155	0.477	1.324
Tetracyclines	0.069	0.155	0.057	1.069
Aminoglycosides	0.132	–^*^	0.210	–^*^
Amphenicoles	0.054	–^*^	0.098	–^*^
Macrolides	0.170	0.145	0.303	0.862
Cephalosporins, 3rd and 4th generation	0.061	–^*^	0.112	–^*^
Fluoroquinolones	0.122	–^*^	0.194	–^*^
Polymyxins	–^*^	0.189	–^*^	–^*^
Total	1.088	0.889	1.485	3.942

* *No products authorized for the combination of class, species and application route*.

Finally, the evolution of the number of potential ACDs per animal for HPCIAs is presented in Table 4. For macrolides used in pigs, a decrease of 22.0% for products sold as premixes was attenuated by a corresponding increase of 11.8% for injectables. This pattern was even more evident in calves where a reduction of 27.1% for premixes was almost completely compensated by an increase of 25.7% in injectables. With respect to the other two classes of HPCIAs, sales of products containing fluoroquinolones remained stable for pigs (−1.5%) and an increase of 6.4% was observed for the number of potential ACDs per animal in calves. Courses with cephalosporins of the third and fourth generations showed a comparable decrease in pigs (−16.4%) and calves (−15.7%).

**Table 4:** Number of estimated ACDs per animal, macrolides, fluoroquinolones and cephalosporins 3rd and 4th generation, for pigs and calves, by administration route.

	**2011**	**2012**	**2013**	**2014**	**2015**
**PIGS**					
Macrolides, premix	0.186	0.199	0.191	0.160	0.145
Macrolides, injections	0.152	0.153	0.171	0.165	0.170
Fluoroquinolones^*^	0.128	0.115	0.142	0.136	0.126
Cephalosporins^*^	0.073	0.062	0.064	0.067	0.061
**CALVES**					
Macrolides, premix	1.183	1.087	0.998	0.891	0.862
Macrolides, injections	0.241	0.239	0.291	0.287	0.303
Fluoroquinolones^*^	0.156	0.145	0.171	0.165	0.166
Cephalosporins^*^	0.133	0.123	0.121	0.119	0.112

* *Only available as injections*.

## Discussion

This is the first study at national level using the ACD concept applied to sales of antimicrobials with the objective of extrapolating the number of potentially treated pigs and calves in Switzerland. Sales of antimicrobials for the veterinary medicine are published at national level since 2005. So far, these data represent the only available source of exhaustive antimicrobial consumption data at national level. Sales figures may allow for the recognition of trends, but the lack of information on potency, dose, duration of treatment and repartition per species strongly limits their usefulness. The indicator ACD may therefore help to bridge that gap. Calculation of ACD and repartition of quantities for products authorized for more than one species would not be possible without making assumptions, which might influence the results. The first assumption relates to the weight of the animals. The standard weight has an impact on both the calculation of the species repartition and the ACD indicator itself. The impact of using different weights is a topic beyond the scope of this study and the impact on calculations has been studied elsewhere (27–29). In this study we used weights at treatment as close as possible to the Swiss reality. This should provide the best fitting results, and also guarantees future reproducibility of the method and comparison of results, as these weights are likely to be used when quantifying Swiss antimicrobial consumption both at national and international level. This approach is comparable to the one chosen by the ESVAC project.

The method used to stratify antimicrobial consumption by the production types included in the study has some potential bias. As it is based on the total biomass of each animal category, the resulting estimates are highly dependent on the animal demographics and the animal average weight used. This might not always be a representative surrogate of the product repartition by each category. As a reliable repartition is generated by data collected on actual usage, and such data are currently not available in Switzerland, we chose an alternative that was applicable at product level that would deliver reproducible results over the years and be as accurate as possible. Carmo et al. (21) have compared three different methods to determine species repartition of antimicrobials. The longitudinal study extrapolation method (based on field data) was not applicable at single product level due to the requirement for minimum, mode, and maximum starting values. The biomass distribution was shown to be the method providing the closest results to the extrapolation based on field data, thereby increasing our confidence on the pertinence of the approach we applied. The two main drawbacks of this method are the dependence on defined average weights and country specific animal demographics. However, the method, limited by the data available in the current Swiss context, provides a first insight into antimicrobial consumption patterns in different species/production types. In the future, the data collection system IS-ABV (description available under http://www.aacting.org/matrix/is-abv/?lid=1447) shall provide further insights into these patterns, as well as a basis for comparison with the results from the method and its potential biases. To make our extrapolations as comparable as possible with other projects, we used the same standard weights as in the ESVAC project (12).

It must also be noticed that the denominators of the indicators presented were based on the number of slaughtered animals only. The weights used for the calculation of the biomass were likely weights at treatment as defined in the ESVAC Project (12). The use of such a calculation might hinder direct comparisons with other studies and should be taken into consideration when benchmarking these results. When using the biomass as a denominator, the result should be interpreted as an indicator for the amount of active ingredient used per kg of animal produced. Likewise, the therapeutic intensity indicates the average number of ACDs per animal produced/slaughtered.

Both a high proportion of heavier animals like cows or, alternatively, a high treatment intensity in a species of lower biomass are examples of how animal demographics can bias the results of the stratification approach based on the biomass. The repartition across species is mainly influenced by national production structures. In Switzerland, dairy production is an important agricultural sector and therefore dairy cows make a high proportion of the food producing animals' sector (15). Cows represented 49% of the total biomass in the year 2015 and this high proportion leads to an underestimation of the repartition of sales for pigs or calves. This primarily affects the repartition of aminoglycosides and cephalosporins of the third generation, which are antimicrobials frequently used in the treatment of dairy cows. The calculated numbers of ACDs per animal for these classes presented in Table 4 are, therefore, an underestimation. Within the same species, biomass repartition could have been used to estimate the use of antimicrobials in different production stages of pigs. However, using piglets, weaners and fattening pigs produced during 1 year introduces the bias of counting a significant but undefined proportion of the animals two or three times. As sales data were only available for one full year, we therefore chose to base our repartition, as well as the denominator for the treatment intensity, on the number of pigs slaughtered during the same year. This indicator is used in this study as a surrogate for all pig production stages.

As the numbers of ACDs represent an extrapolation of usage data based on sales figures, they follow the latter closely. The downward trends in sales is mirrored by the treatment numbers of both calves and pigs. However, differences become evident as soon as additional factors like application route are taken into account. The repartition for pigs in the year 2015 shows that 18% of the active ingredients were used parenterally when based on quantity, whereas they represented 51% of the treatments when using ACDs. The main reason for this difference lies in the potency of the active ingredients: antimicrobials are used parenterally with a lower dose as there is no loss of active ingredient compared to the lower bioavailability following oral application. Another possible reason is the use of more than one ACD for parenterally applied combination products as 12 of 71 injectable products investigated were combinations of two active ingredients. Whereas, this approach can be disputed as it shows a higher number of “treatments,” we think that the use of ACDs is better suited to test for associations between antimicrobial use and resistance.

Converting sales of antimicrobials to number of treatments per animal allows detection of trends that would not be obvious when only assessing the quantity of active ingredients sold. Macrolides used to treat calves provide a good example: our results show a clear shift from the oral application in form of premixes toward an increase in the use of injectables. One possible explanation is the increasing availability of macrolide antibiotics with a long duration of action, e.g., tulathromycin, tildipirosin, and gamithromycin. Such active compounds combine the easy use of a single application with a long action. Moreover, for parenteral applications, both time to maximal concentration and maintenance of active levels are not influenced by the appetite of the animals, therefore guaranteeing the adequate treatment of sick animals with reduced appetite. On the negative side, studies about macrolides used in human medicine convincingly showed a higher level of resistance selection for longer acting molecules (30).

Our results show a strong difference in the extrapolated usage of antimicrobials in pigs and calves. This cannot be explained by a single factor as the administration of antimicrobials is driven by medical, economic and also psychosocial factors. Crowding effect, stress during transport of very young, not yet immunocompetent animals, partially inadequate colostrum feeding and less than ideal stable climate are among the factors favoring respiratory problems in calves (31, 32). In the swine industry, some of the abovementioned factors also exist, but the structure and management of pig production limits the risks. Management practices like all-in-all-out including disinfection between the batches or integrated production from piglet to finisher can strongly help to reduce antimicrobial usage. In pigs, there are two main periods at risk for treatment with antimicrobials: the first at weaning with around 12 kg body weight and the second at around 25 to 29 kg body weight (25, 33). In pigs, diarrhea is one of the leading indications for treatment. This is a very unspecific symptom with many different causes, including not only bacterial but also dietary or viral origins. In this context, the availability of vaccines against both circovirus and *Lawsonia intracellularis* infections in the years 2008 to 2010 contributed to the reduction of diarrheal symptoms, and hence, the rather indiscriminate use of antibiotics to treat such symptoms. For calves, respiratory diseases are much more multifactorial and the introduction of various vaccines (against bovine respiratory syncytial, parainfluenza or corona virus) seems not to have had the same positive effect as in the pig industry.

Several factors hinder a proper comparison of our results with previously published data. To the best of our knowledge, this is the first time that the ACD indicator is used at national level in Switzerland. As a matter of fact, its use is not currently widespread in other countries, with the exception of France where it was developed. However, the comparison with French data is difficult. No publication presents the French antimicrobial consumption using ACDs per animal and year as an indicator. The French indicator for exposure to antimicrobials is ALEA [Animal Level of Exposure to Antimicrobials; (16)]. It is obtained by dividing the effectively treated biomass by the total biomass of the same species. The global ALEA calculated for the year 2015 in France was 0.488 and represented a decrease of 20.1% compared to 2011. Another difficulty is the use of different production categories and standard weights at treatment. For pigs, the French system uses weights up to 350 kg for a specific category of sows and the average for the pig population is set at 105 kg. This is 3.62 times higher than the standard weight at treatment of 29 kg identified in previous Swiss studies and used here. The differences in the standard weights at treatment also explain the discrepancies in the antimicrobial consumption for France published, for the same year, in the ESVAC report (107 mg/PCU) and in the ANSES report (47 mg/kg). Due to the differences in weight and in the categories, and the difficulties in making assumptions and extrapolations, we decided not the compare our figures to the French ones.

Our data can only be compared with countries where calves are reared for the production of veal meat. Besides France and Belgium (for which country we could not find adequate data for comparison), this production system also exists in The Netherlands. The available report for the year 2015 (34) uses indicators differing from the ones in the present study but still shows a higher treatment intensity in calves compared to pigs. This is in line with the present study, where the antimicrobial use was 2.77-fold higher in calves than in pigs.

Both examples clearly illustrate the need to harmonize methodologies at international level in order to discuss data collected in different countries. Such discussions currently take place within the AACTNG network (www.aacting.org).

## Conclusion

This first study of the number of treatments of pigs and calves extrapolated from yearly sales shows both similarities and differences between the two species under consideration. Whereas, the sales by species and the number of extrapolated treatments both decreased in a similar way, the difference in the number of treatments per animal between pigs and calves differed over the years under investigation. Given that the applied method is based on the extrapolation of sales figures, a similar decrease at species level was to be expected. However, the use of course doses allows to further investigate trends in the patterns of antimicrobial treatments. In our study, this was very clear for the class of macrolides, for which the decreases in oral use were partly (pigs) or completely (calves) compensated by the application of long acting injectables. We, therefore, recommend the use of extrapolated treatment numbers when no exhaustive collection of usage data is in place. The concept of ACDs can also complement the collection of antimicrobial consumption data at species level allowing for their validation using sales data.

## Data Availability Statement

All datasets generated for this study are included in the manuscript/Supplementary Files.

## Author Contributions

RS did all the calculations presented in this work. LC helped with the repartition of sales between species, expert advice and biomass distribution. Raw sales data and advice regarding their use was provided by DH. The study was designed by CM and supervised by KE and HN.

### Conflict of Interest

The authors declare that the research was conducted in the absence of any commercial or financial relationships that could be construed as a potential conflict of interest.
