# Supplementary material for: Extrapolating Antibiotic Sales to Number of Treated Animals: Treatments in Pigs and Calves in Switzerland, 2011–2015
Source: Front Vet Sci. 2019 Sep 20;6:318. doi: 10.3389/fvets.2019.00318 (PMC6763737; doi:10.3389/fvets.2019.00318)
Supplement: Supplementary file 1 [file Table_1.DOCX]

Supplementary Material

**
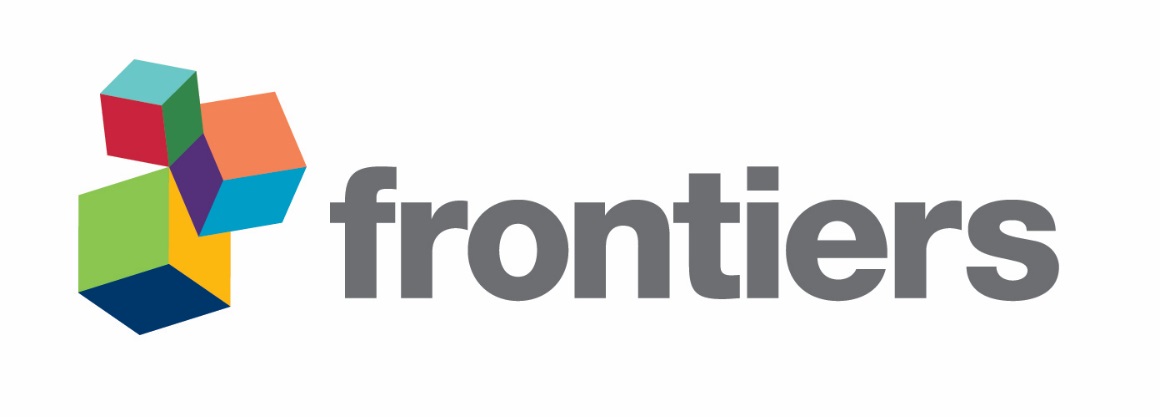
**

**Supplementary Table 1.** Weights at treatment and number of animals used to calculate the biomass repartition

| Species | 2011 | 2012 | 2013 | 2014 | 2015 | Weight at treatment^1^ |
| --- | --- | --- | --- | --- | --- | --- |
| Dairy cows | 699’947 | 705’642 | 703’489 | 705’371 | 701’172 | 425 |
| Calves^2^ | 261’308 | 256’471 | 252’118 | 251’509 | 236’343 | 140 |
| Sows | 135’715 | 128’367 | 125’219 | 123’639 | 122’542 | 220 |
| Pigs^2^ | 2’839’106 | 2’773’726 | 2’689’576 | 2’751’721 | 2’753’256 | 65 |
| Sheep | 424’018 | 417’274 | 409’493 | 402’272 | 346’549 | 75 |
| Goats | 82’812 | 84’732 | 84’541 | 84’654 | 71’249 | 65^3^ |
| Horses | 57’246 | 58’031 | 57’243 | 57’200 | 55’455 | 400 |
| Dogs | 526’977 | 531’135 | 536’258 | 506’000 | 508’000^4^ | 20 |
| Cats | 1’500’000 | 1’487’000 | 1’500’000 | 1’487’000 | 1’487’000 | 5 |

^1^Weights according to ESVAC report (EMA, 2011)

^2^Slaughtered numbers. All other species represented by live numbers.

^3^From Montforts, 2006

^4^Mean of 2014 and 2016 (510’000) as number not available at time of study.
